# Complete Genome Sequence of *Collinsella aerofaciens* Isolated from the Gut of a Healthy Indian Subject

**DOI:** 10.1128/genomeA.01361-17

**Published:** 2017-11-22

**Authors:** Satyabrata Bag, Tarini Shankar Ghosh, Bhabatosh Das

**Affiliations:** Molecular Genetics Laboratory, Centre for Human Microbial Ecology, Translational Health Science and Technology Institute, NCR Biotech Science Cluster, Faridabad, India

## Abstract

*Collinsella aerofaciens*, a rod-shaped nonmotile obligate anaerobe, is the most abundant actinobacterium in the gastrointestinal tract of healthy humans. An altered abundance of *C. aerofaciens* may be linked with several health disorders, including irritable bowel syndrome. In the present study, we report the complete genome sequence of *C. aerofaciens* strain indica.

## GENOME ANNOUNCEMENT

The bacterium *Collinsella aerofaciens* is well known for its ability to ferment a range of plant and animal origin carbohydrates and for producing H_2_, ethanol, short-chain fatty acids, and lactate in the human colon (1). *C. aerofaciens* is the major utilizer of lactose in the human colon. Several studies demonstrated that *Collinsella* and *Bifidobacterium* can modify the host bile acids to modulate the virulence and pathogenicity of enteric pathogens (2). Recently, it was reported that an altered abundance of *Collinsella* may also influence host plasma cholesterol levels (3). To understand the importance of *C. aerofaciens* in health and disease, it is important to explore its genomic repertoire and identify functions that potentially influence host physiology.

In the present study, *C. aerofaciens* strain indica was isolated from a fecal sample of a healthy adult Indian subject. An approximately 500-mg fecal sample was resuspended in 1 ml of phosphate-buffered saline (PBS), diluted serially in the same buffer, and plated on a prereduced Trypticase soy agar plate (pH 7.3) supplemented with 5% (vol/vol) defibrinated sheep blood. Bacterial cells were spread over the surface of the plates using four or five glass beads (3.00 mm). Plates were incubated for 48 h at 37°C in an anaerobic workstation (Whitley DG250) filled with 80% N_2_, 10% CO_2_, and 10% H_2_. A single colony of *C. aerofaciens* was grown in 5 ml of tryptic soy broth (TSB) for 48 h. Growth of the cells in TSB was monitored by spectrophotometer. *C. aerofaciens* cells were harvested from 2 ml of culture by centrifugation (8,000 × *g* for 3 min), and the genomic DNA was extracted by the THSTI method (4).

The whole-genome sequencing of *C. aerofaciens* was carried out by utilizing 2 different DNA sequencing platforms, those from Illumina (HiSeq 2500 system) and Oxford Nanopore Technologies (MinION). The SPAdes tool was used to assemble error-corrected long Nanopore reads and Illumina reads, which generated a single contig. The assembled complete genome sequence of *C. aerofaciens* was evaluated by Sanger sequencing. The complete genome of *C. aerofaciens* strain indica is 2,306,349 bp in length, with 60.1% GC content.

The analysis of the 2.30-Mb genome sequence of *C. aerofaciens* identified 1,995 genes, including 75 RNA-encoding genes. The genome has 276 subsystems and is enriched with protein (231 open reading frames [ORFs]), carbohydrate (226 ORFs), amino acid (200 ORFs), cofactor, and vitamin (102 ORFs) metabolic functions. The *C. aerofaciens* genome is also enriched with fatty acid, lipid, and isoprenoid metabolic functions (56 ORFs). However, the *C. aerofaciens* genome has several antibiotic resistance genes, such as β-lactamase, tetracycline resistance and ribosome protection functions, and multidrug resistance efflux pumps, but no gene was detected for the subcategory of virulence, pathogenicity, and disease development. The complete genome sequence of *C. aerofaciens* strain indica will contribute to a better understanding of the biology of the commensal and the molecular basis of its dominance in the gut of Indian subjects.

### Accession number(s).

The whole-genome shotgun project has been deposited at DDBJ/ENA/GenBank under the accession number CP024160. The version described in this paper is CP024160.1.
